# *N*-(2-Amino-4,6-dihydroxypyrimidin-5-yl)acetamide dihydrate

**DOI:** 10.1107/S1600536811034441

**Published:** 2011-08-31

**Authors:** Xiao-Min Zhang, Hui-Liang Zhou, Qi-Lin Hu

**Affiliations:** a College of Chemistry and Chemical Engineering, Ningxia University, Yinchuan 750021, Ninxia, People’s Republic of China

## Abstract

The title compound, C_6_H_8_N_4_O_3_·2H_2_O, which crystallized as a dihydrate, has two almost planar segments *viz.*  the pyrimidine ring and the C—N—C(=O)—C group [maxmum deviations of 0.020 (2) and 0.014 (2) Å, respectively], with a dihedral angle of 87.45°. In the crystal, the components are linked by O—H⋯O and N—H⋯O hydrogen bonds.

## Related literature

For the biological properties of pyrimidine compounds see: Marchal *et al.* (2010 ▶); Giandinoto *et al.* (1996 ▶); Sun *et al.* (2006 ▶). For related structures, see: Glidewell *et al.* (2003 ▶); Nakayama *et al.* (2004 ▶); Quesada *et al.* (2004 ▶); Hockova *et al.* (2003 ▶).
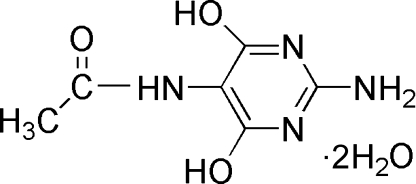


## Experimental

### 

#### Crystal data

C_6_H_8_N_4_O_3_·2H_2_O
*M* *_r_* = 220.20
Monoclinic, 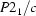

*a* = 9.5501 (12) Å
*b* = 12.2161 (13) Å
*c* = 8.5324 (8) Å
β = 98.708 (1)°
*V* = 983.96 (19) Å^3^
*Z* = 4
Mo *K*α radiation
μ = 0.13 mm^−1^
*T* = 298 K
0.23 × 0.15 × 0.10 mm

#### Data collection

Bruker SMART CCD area-detector diffractometer
Absorption correction: multi-scan (*SADABS*; Bruker, 2002 ▶) *T*_min_ = 0.971, *T*_max_ = 0.987
5002 measured reflections
1727 independent reflections
1082 reflections with *I* > 2σ(*I*)
*R*_int_ = 0.039

#### Refinement

*R*[*F* ^2^ > 2σ(*F* ^2^)] = 0.042
*wR*(*F* ^2^) = 0.120
*S* = 0.85
1727 reflections
136 parameters
H-atom parameters constrained
Δρ_max_ = 0.20 e Å^−3^
Δρ_min_ = −0.26 e Å^−3^

### 

Data collection: *SMART* (Bruker, 2002 ▶); cell refinement: *SMART*; data reduction: *SAINT* (Bruker, 2002 ▶); program(s) used to solve structure: *SHELXS97* (Sheldrick, 2008 ▶); program(s) used to refine structure: *SHELXL97* (Sheldrick, 2008 ▶); molecular graphics: *SHELXTL* (Sheldrick, 2008 ▶); software used to prepare material for publication: *SHELXTL*.

## Supplementary Material

Crystal structure: contains datablock(s) I, global. DOI: 10.1107/S1600536811034441/fl2354sup1.cif

Structure factors: contains datablock(s) I. DOI: 10.1107/S1600536811034441/fl2354Isup2.hkl

Supplementary material file. DOI: 10.1107/S1600536811034441/fl2354Isup3.cml

Additional supplementary materials:  crystallographic information; 3D view; checkCIF report

## Figures and Tables

**Table 1:** Hydrogen-bond geometry (Å, °)

*D*—H⋯*A*	*D*—H	H⋯*A*	*D*⋯*A*	*D*—H⋯*A*
N1—H1⋯O1^i^	0.86	1.98	2.810 (2)	164
N2—H2⋯O2^ii^	0.86	2.00	2.836 (2)	163
N3—H3*A*⋯O5^ii^	0.86	1.95	2.803 (2)	172
N3—H3*B*⋯O4^i^	0.86	1.98	2.843 (2)	178
N4—H4⋯O2^iii^	0.86	2.27	3.115 (2)	170
O4—H4*A*⋯O1	0.85	1.90	2.717 (2)	159
O4—H4*B*⋯O3^iv^	0.85	1.96	2.812 (2)	176
O5—H5*A*⋯O2	0.85	1.95	2.716 (2)	150
O5—H5*B*⋯O3^iii^	0.85	1.97	2.814 (2)	174

Symmetry codes: (i) 
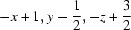
; (ii) 
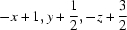
; (iii) 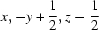; (iv) 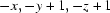.

**Fig. 1.**
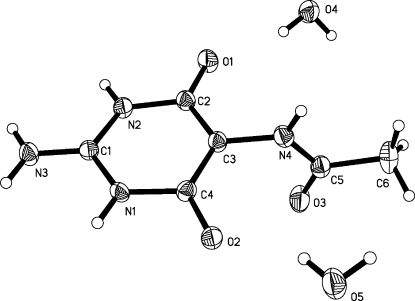
The molecular structure of the title compound, showing 30% displacement ellipsoids for the non-hydrogen atoms. Hydrogen atoms are drawn as spheres of arbitrary radius.

**Fig. 2.**
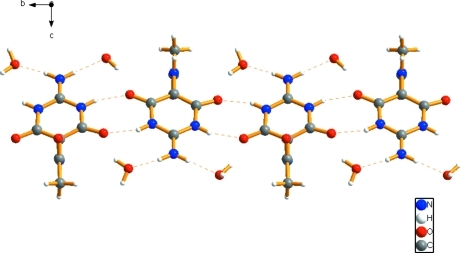
One-dimensional chain of the title compound. Hydrogen bonds are shown as dashed lines.

**Fig. 3.**
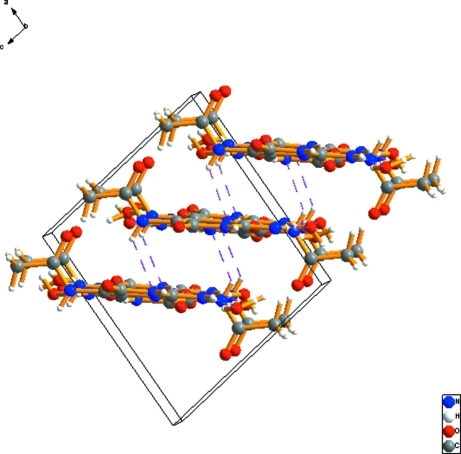
The molecular packing of the title compound, viewed along the b axis. Intermolecular hydrogen bonds are indicated by dashed lines.

## supplementary crystallographic information

### Comment

Pyrimidine and its derivatives are important targets for drug discovery having attracted much attention for their biological activities and molecular structures (Sun *et al.*, (2006)). Research findings indicate that the pyrimidine derivatives are associated with diverse pharmacological activities, such as antifungal, antibacterial, pesticidal, analgesic, and antitumor (Giandinoto *et al.*; (1996); Nakayama *et al.*, (2004); Hockova *et al.*, (2003)). The present X-ray crystal structure analysis was undertaken in order to study the stereochemistry and crystal packing of the title compound (I).

As shown in Fig 1, the title compound which crystallized as a dihydrate is composed of two planar segments. One segment is a pyrimidine ring, which (C1, N2, C2, C3, C4, N1), and the other segment contains C3, N4, C6, C5 and O3. The dihedral angle between the two planar segments is 87.45 °. (I)crystallized in the keto form.

The molecule exhibits O..H···O hydrogen bonding with the water molecules and intermoleculer N-H···O hydrogen bonding between the pyrimidine moieties (Table 1). The chains  formed by the the N—H···O hydrogen bonds can be seen in Fig. 2. The crystal structure is also aggregated into a three-dimensional framework *via*  further N—H···O interactions (Fig.3).

### Experimental

A mixture of guanidine hydrochloride (2.04 g, 4 mmol) and diethyl acetylaminomalonate (5.0 g, 2 mmol) were reacted in 36 ml sodium ethylate at 358 K for 5 h. Then the product was disolved in water with proper pH adjustment (3–4) by HCl. After filtering and drying, the crystalline product of the title compound was collected by recrystallization at room temperature in 10% HCl(10 ml).

### Refinement

All the H atoms were placed in geometrically idealized positions and constrained to ride on their parent atoms, with O—H distances of 0.85 Å, N—-H distances of 0.86 Å, C—H distances of 0.93–0.97 Å and *U*_iso_(H) = 1.2–1.5*U*_eq_ of the parent atom.

### Crystal data

**Table:**

C_6_H_8_N_4_O_3_·2H_2_O	*F*(000) = 464
*M**_r_* = 220.20	*D*_x_ = 1.486 Mg m^−^^3^
Monoclinic, *P*2_1_/*c*	Mo *K*α radiation, λ = 0.71073 Å
*a* = 9.5501 (12) Å	Cell parameters from 1089 reflections
*b* = 12.2161 (13) Å	θ = 2.7–26.2°
*c* = 8.5324 (8) Å	µ = 0.13 mm^−^^1^
β = 98.708 (1)°	*T* = 298 K
*V* = 983.96 (19) Å^3^	Cuboid, colorless
*Z* = 4	0.23 × 0.15 × 0.10 mm

### Data collection

**Table:**

Bruker SMART CCD area-detector diffractometer	1727 independent reflections
Radiation source: fine-focus sealed tube	1082 reflections with *I* > 2σ(*I*)
graphite	*R*_int_ = 0.039
Detector resolution: 0 pixels mm^-1^	θ_max_ = 25.0°, θ_min_ = 2.2°
φ and ω scans	*h* = −11→11
Absorption correction: multi-scan (*SADABS*; Bruker, 2002)	*k* = −14→13
*T*_min_ = 0.971, *T*_max_ = 0.987	*l* = −9→10
5002 measured reflections	

### Refinement

**Table:**

Refinement on *F*^2^	Primary atom site location: structure-invariant direct methods
Least-squares matrix: full	Secondary atom site location: difference Fourier map
*R*[*F*^2^ > 2σ(*F*^2^)] = 0.042	Hydrogen site location: inferred from neighbouring sites
*wR*(*F*^2^) = 0.120	H-atom parameters constrained
*S* = 0.85	*w* = 1/[σ^2^(*F*_o_^2^) + (0.0641*P*)^2^ + 0.2581*P*] where *P* = (*F*_o_^2^ + 2*F*_c_^2^)/3
1727 reflections	(Δ/σ)_max_ < 0.001
136 parameters	Δρ_max_ = 0.20 e Å^−^^3^
0 restraints	Δρ_min_ = −0.26 e Å^−^^3^

### Special details

**Table:**

Geometry. All e.s.d.'s (except the e.s.d. in the dihedral angle between two l.s. planes) are estimated using the full covariance matrix. The cell e.s.d.'s are taken into account individually in the estimation of e.s.d.'s in distances, angles and torsion angles; correlations between e.s.d.'s in cell parameters are only used when they are defined by crystal symmetry. An approximate (isotropic) treatment of cell e.s.d.'s is used for estimating e.s.d.'s involving l.s. planes.
Refinement. Refinement of *F*^2^ against ALL reflections. The weighted *R*-factor *wR* and goodness of fit *S* are based on *F*^2^, conventional *R*-factors *R* are based on *F*, with *F* set to zero for negative *F*^2^. The threshold expression of *F*^2^ > σ(*F*^2^) is used only for calculating *R*-factors(gt) *etc*. and is not relevant to the choice of reflections for refinement. *R*-factors based on *F*^2^ are statistically about twice as large as those based on *F*, and *R*- factors based on ALL data will be even larger.

### Fractional atomic coordinates and isotropic or equivalent isotropic displacement parameters (Å^2^)

**Table:**

	*x*	*y*	*z*	*U*_iso_*/*U*_eq_	
O4	0.21459 (17)	0.66228 (13)	0.37653 (19)	0.0498 (5)	
N1	0.51891 (18)	0.27137 (14)	0.8076 (2)	0.0336 (5)	
H1	0.5490	0.2108	0.8520	0.040*	
N2	0.53066 (18)	0.45908 (14)	0.7961 (2)	0.0350 (5)	
H2	0.5664	0.5196	0.8351	0.042*	
N3	0.68269 (19)	0.36480 (15)	0.9877 (2)	0.0403 (5)	
H3A	0.7194	0.4256	1.0247	0.048*	
H3B	0.7129	0.3038	1.0305	0.048*	
N4	0.25806 (19)	0.36468 (14)	0.4697 (2)	0.0364 (5)	
H4	0.2839	0.3634	0.3774	0.044*	
O1	0.38963 (16)	0.55753 (12)	0.60969 (18)	0.0443 (5)	
O2	0.35840 (16)	0.17282 (12)	0.64300 (17)	0.0408 (4)	
O3	0.07419 (17)	0.37021 (14)	0.60637 (19)	0.0526 (5)	
H4A	0.2598	0.6153	0.4382	0.063*	
H4B	0.1272	0.6500	0.3778	0.063*	
O5	0.1825 (2)	0.06285 (14)	0.4167 (2)	0.0641 (6)	
H5A	0.2207	0.1168	0.4697	0.077*	
H5B	0.1527	0.0876	0.3245	0.077*	
C1	0.5807 (2)	0.36517 (17)	0.8661 (2)	0.0313 (5)	
C2	0.4238 (2)	0.46472 (18)	0.6633 (2)	0.0327 (5)	
C3	0.3655 (2)	0.36483 (17)	0.6053 (2)	0.0320 (5)	
C4	0.4087 (2)	0.26646 (18)	0.6789 (2)	0.0314 (5)	
C5	0.1185 (2)	0.36648 (18)	0.4780 (3)	0.0377 (6)	
C6	0.0201 (3)	0.3621 (2)	0.3226 (3)	0.0566 (8)	
H6A	−0.0314	0.2944	0.3152	0.085*	
H6B	0.0743	0.3670	0.2367	0.085*	
H6C	−0.0452	0.4222	0.3166	0.085*	

### Atomic displacement parameters (Å^2^)

**Table:**

	*U*^11^	*U*^22^	*U*^33^	*U*^12^	*U*^13^	*U*^23^
O4	0.0423 (10)	0.0475 (11)	0.0557 (11)	−0.0018 (8)	−0.0048 (8)	0.0086 (8)
N1	0.0395 (11)	0.0234 (10)	0.0348 (10)	0.0006 (8)	−0.0042 (9)	0.0052 (8)
N2	0.0390 (11)	0.0231 (10)	0.0398 (11)	−0.0015 (8)	−0.0039 (9)	−0.0026 (8)
N3	0.0443 (12)	0.0300 (10)	0.0418 (11)	0.0004 (9)	−0.0090 (9)	−0.0010 (9)
N4	0.0418 (12)	0.0348 (11)	0.0312 (10)	0.0005 (9)	0.0009 (8)	−0.0002 (8)
O1	0.0548 (11)	0.0217 (9)	0.0508 (10)	0.0016 (7)	−0.0104 (8)	0.0031 (7)
O2	0.0472 (10)	0.0251 (9)	0.0452 (9)	−0.0033 (7)	−0.0092 (7)	0.0004 (7)
O3	0.0398 (10)	0.0727 (13)	0.0433 (10)	−0.0004 (9)	−0.0004 (8)	−0.0034 (9)
O5	0.0867 (14)	0.0427 (11)	0.0521 (11)	−0.0023 (10)	−0.0241 (10)	−0.0001 (9)
C1	0.0329 (12)	0.0273 (12)	0.0328 (11)	0.0001 (10)	0.0020 (10)	−0.0005 (10)
C2	0.0335 (12)	0.0301 (13)	0.0336 (12)	0.0022 (10)	0.0019 (10)	0.0008 (10)
C3	0.0344 (12)	0.0277 (12)	0.0319 (12)	0.0004 (10)	−0.0017 (10)	−0.0008 (10)
C4	0.0316 (12)	0.0290 (13)	0.0331 (12)	−0.0020 (10)	0.0029 (10)	−0.0036 (10)
C5	0.0427 (15)	0.0293 (12)	0.0385 (13)	0.0016 (11)	−0.0025 (11)	−0.0034 (10)
C6	0.0507 (16)	0.0645 (19)	0.0473 (15)	0.0095 (13)	−0.0159 (13)	−0.0124 (13)

### Geometric parameters (Å, °)

**Table:**

O4—H4A	0.8504	N4—H4	0.8600
O4—H4B	0.8494	O1—C2	1.247 (2)
N1—C1	1.350 (3)	O2—C4	1.260 (2)
N1—C4	1.403 (2)	O3—C5	1.234 (3)
N1—H1	0.8600	O5—H5A	0.8501
N2—C1	1.347 (3)	O5—H5B	0.8501
N2—C2	1.407 (2)	C2—C3	1.400 (3)
N2—H2	0.8600	C3—C4	1.389 (3)
N3—C1	1.312 (3)	C5—C6	1.505 (3)
N3—H3A	0.8600	C6—H6A	0.9600
N3—H3B	0.8600	C6—H6B	0.9600
N4—C5	1.345 (3)	C6—H6C	0.9600
N4—C3	1.426 (3)		
			
H4A—O4—H4B	106.3	O1—C2—N2	117.21 (19)
C1—N1—C4	124.06 (18)	C3—C2—N2	116.30 (19)
C1—N1—H1	118.0	C4—C3—C2	121.27 (19)
C4—N1—H1	118.0	C4—C3—N4	119.58 (19)
C1—N2—C2	124.33 (18)	C2—C3—N4	119.14 (19)
C1—N2—H2	117.8	O2—C4—C3	126.82 (19)
C2—N2—H2	117.8	O2—C4—N1	116.23 (19)
C1—N3—H3A	120.0	C3—C4—N1	116.95 (19)
C1—N3—H3B	120.0	O3—C5—N4	121.5 (2)
H3A—N3—H3B	120.0	O3—C5—C6	122.1 (2)
C5—N4—C3	123.67 (19)	N4—C5—C6	116.4 (2)
C5—N4—H4	118.2	C5—C6—H6A	109.5
C3—N4—H4	118.2	C5—C6—H6B	109.5
H5A—O5—H5B	105.9	H6A—C6—H6B	109.5
N3—C1—N2	121.66 (19)	C5—C6—H6C	109.5
N3—C1—N1	121.37 (19)	H6A—C6—H6C	109.5
N2—C1—N1	116.96 (17)	H6B—C6—H6C	109.5
O1—C2—C3	126.5 (2)		
			
C2—N2—C1—N3	178.5 (2)	C5—N4—C3—C4	85.7 (3)
C2—N2—C1—N1	−2.9 (3)	C5—N4—C3—C2	−93.2 (3)
C4—N1—C1—N3	179.4 (2)	C2—C3—C4—O2	175.6 (2)
C4—N1—C1—N2	0.9 (3)	N4—C3—C4—O2	−3.3 (3)
C1—N2—C2—O1	−178.93 (19)	C2—C3—C4—N1	−3.7 (3)
C1—N2—C2—C3	1.6 (3)	N4—C3—C4—N1	177.38 (19)
O1—C2—C3—C4	−177.5 (2)	C1—N1—C4—O2	−177.07 (19)
N2—C2—C3—C4	1.9 (3)	C1—N1—C4—C3	2.4 (3)
O1—C2—C3—N4	1.4 (3)	C3—N4—C5—O3	1.2 (3)
N2—C2—C3—N4	−179.17 (18)	C3—N4—C5—C6	−178.0 (2)

### Hydrogen-bond geometry (Å, °)

**Table:**

*D*—H···*A*	*D*—H	H···*A*	*D*···*A*	*D*—H···*A*
N1—H1···O1^i^	0.86	1.98	2.810 (2)	164.
N2—H2···O2^ii^	0.86	2.00	2.836 (2)	163.
N3—H3A···O5^ii^	0.86	1.95	2.803 (2)	172.
N3—H3B···O4^i^	0.86	1.98	2.843 (2)	178.
N4—H4···O2^iii^	0.86	2.27	3.115 (2)	170.
O4—H4A···O1	0.85	1.90	2.717 (2)	159.
O4—H4B···O3^iv^	0.85	1.96	2.812 (2)	176.
O5—H5A···O2	0.85	1.95	2.716 (2)	150.
O5—H5B···O3^iii^	0.85	1.97	2.814 (2)	174.

Symmetry codes: (i) −*x*+1, *y*−1/2, −*z*+3/2; (ii) −*x*+1, *y*+1/2, −*z*+3/2; (iii) *x*, −*y*+1/2, *z*−1/2; (iv) −*x*, −*y*+1, −*z*+1.
